# Letter to editor: The evidence-base for the management of flexor tendon injuries of the hand: Review

**DOI:** 10.1016/j.amsu.2020.03.014

**Published:** 2020-05-16

**Authors:** Jing Chen, Zhang Jun Pan

**Affiliations:** a Department of Hand Surgery, Affiliated Hospital of Nantong University, Nantong, Jiangsu, China; b Department of Hand Surgery, People's Hospital of Yixin, Wuxi, Jiangsu, China

Dear editor,

We read with great interest of the article, ‘The evidence-base for the management of flexor tendon injuries of the hand: Review’ [1] and thank the authors for providing readers the evidence. Much of the information in the review is educational.

We found the authors missed to include a key technique to ensure a successful tendon repair in zone 2: venting of the critical pulleys. The authors only had one sentence about this key technique and the statement is imprecise. The authors stated “it is important to preserve the A2 and A4 pulleys to prevent bowstringing although the A2 can be partially vented if necessary, but this ought to be done meticulously as venting has been associated with increased glide resistance and reduced finger range of motion” with the reference to a paper published 15 years ago. The practice has changed greatly over past 15 years, and venting of the critical pulley is as important as implementing a strong repair technique such as a 6-strand repairs [[2], [3], [4], [5], [6], [7], [8], [9], [10], [11]]. All publications of clinical outcomes in zone 2 in recent years indicate such as need [5,[12], [13], [14], [15], [16], [17]]. The outcomes from our units also indicate the need, besides the reports from Europe and North America [12,13,16]. Our experience indicates if venting is not sufficiently performed, the outcomes will still be unpredictable even a strong repair method is used. We would like to complement the authors’ review with this critical procedure, and alert the readers to this critical technique which has been largely accepted and practiced over the past 20 years.

The mentioning of the need of preserving the A4 pulley in this review is against the updated practice in this field. Absolute need of preservation of the A4 pulley during primary flexor tendon repair is actually a dogma [18], which is now abandoned. It is very clear that when all other annular pulleys are intact, the A4 pulley can be vented to expose the cut tendon ends as well as accommodating the surgical repairs. Recent reports also indicate no clinical consequences after venting both A4 and A3 pulleys at the same time [11], which is sometimes necessary to repair the cut flexor tendons in zone 2A or 2B, as are attested also our own patients.

We would like to mention the changes in early active flexion exercise after primary flexor tendon repair, which was not mentioned specifically in the review. The early active motion has replaced the passive motion regimes in many units over past 20 years [11,13]. The evidence of infrequent (or even no) need of tenolysis after primary tendon repair can be found in all recent reports given in the references with early active finger flexion exercise, in contract to one report [19] with a high tenolysis rate after passive motion exercise. This fact should also brought to attention of the readers.

## Ethical approval

Not applicable.

## Sources of funding

Nothing to declare.

## Author contribution

All authors contributed writing the commentary to the Editor.

## Provenance and peer review

Not commissioned, externally peer reviewed.

## Declaration of competing interest

Nothing to declare.
